# Occupational exposure and radiobiological risk from thyroid radioiodine therapy in Saudi Arabia

**DOI:** 10.1038/s41598-021-93342-1

**Published:** 2021-07-15

**Authors:** H. I. Al-Mohammed, A. Sulieman, Fareed H. Mayhoub, Hassan Salah, Celestino Lagarde, M. Alkhorayef, Ali Aldhebaib, C. Kappas, D. A. Bradley

**Affiliations:** 1 Department of Radiological Sciences, College of Health and Rehabilitation Sciences, Princess Nourah Bint Abdulrahman University, P.O. Box 84428, Riyadh, 11671 Saudi Arabia; 2 Radiology and Medical Imaging Department, College of Applied Medical Sciences, Prince Sattam Bin Abdulaziz University, P.O. Box 422, Alkharj, 11942 Saudi Arabia; 3 King Faisal Specialist Hospital & Research Centre, Riyadh, Saudi Arabia; 4 Nuclear Medicine Department, INAYA Medical Collage, Riyadh, 13541 Saudi Arabia; 5 College of Medical Radiologic Science, Sudan University of Science and Technology, P.O. Box 1908, Khartoum, 11111 Sudan; 6 Department of Radiological Sciences, College of Applied Medical Sciences, King Saud University, P.O. Box 10219, Riyadh, 11433 Saudi Arabia; 7 Department of Physics, Centre for Nuclear and Radiation Physics, University of Surrey, Guildford, GU2 7XH Surrey UK; 8 Radiological Sciences Program, College of Applied Medical Sciences (COMAS), King Saud Bin Abdulaziz University for Health Sciences (KSAU-US), King Abdul Aziz Medical City (KAMC), King Abdullah International Medical Research Centre (KAIMRC), National Guard Health Affairs (NGHA), Riyadh, Kingdom of Saudi Arabia; 9 Department of Medical Physics, Medical School, University of Thessaly, 41110 Larissa, Greece; 10 Centre for Applied Physics and Radiation Technologies, School of Engineering and Technology, Sunway University, Jalan Universiti, 47500 Bandar Sunway Selangor, Malaysia

**Keywords:** Health care, Health occupations, Biological physics

## Abstract

Worldwide, thyroid cancer accounts for some 10% of total cancer incidence, most markedly for females. Thyroid cancer radiotherapy, typically using ^131^I (T_1/2_ 8.02 days; β^−^ max energy 606 keV, branching ratio 89.9%), is widely adopted as an adjunct to surgery or to treat inoperable cancer and hyperthyroidism. With staff potentially receiving significant doses during source preparation and administration, radiation protection and safety assessment are required in ensuring practice complies with international guidelines. The present study, concerning a total of 206 patient radioiodine therapies carried out at King Faisal Specialist Hospital and Research Center over a 6-month period, seeks to evaluate patient and occupational exposures during hospitalization, measuring ambient doses and estimating radiation risk. Using calibrated survey meters, patient exposure dose-rate estimates were obtained at a distance of 30-, 100- and 300 cm from the neck region of each patient. Occupational and ambient doses were measured using calibrated thermoluminescent dosimeters. The mean and range of administered activity (AA, in MBq) for the thyroid cancer and hyperthyroidism treatment groups were 4244 ± 2021 (1669–8066), 1507.9 ± 324.1 (977.9–1836.9), respectively. The mean annual occupational doses were 1.2 mSv, that for ambient doses outside of the isolation room corridors were found to be 0.2 mSv, while ambient doses at the nursing station were below the lower limit of detection. Exposures to staff from patients being treated for thyroid cancer were less compared to hyperthyroidism patients. With a well-defined protocol, also complying with international safety requirements, occupational exposures were found to be relatively high, greater than most reported in previous studies.

## Introduction

In Saudi Arabia, the incidence of thyroid cancer (TC) accounts for 11% of the total cancer incidence, making it the 2nd most common cancer in the country. The incidence of thyroid cancer is greater in females, 77.7%, compared to 22.3% in males^1^ for males alone it is the 8th most common cancer. This incidence is significantly greater than that in the USA, wherein thyroid cancer represents only 2.9% of all malignancies and 4.6% of all female malignancies^2,3^. The median age and range at diagnosis were 39.0 (4.0–95.0) and 44.0 (8.0–95.0) years, for females and males respectively^1^. The incidence of thyroid cancer continues to increase in Saudi Arabia, with a 24% increase in males and a 63% increase among females over the ten-year period up to 2018^1^. For treatment, it has been estimated that about 60% received combined modality treatment consisting of surgery, radiation, and hormonal therapy. Thyroid disease treated with radioiodine (^131^I) includes cancer and non-cancerous diseases such as hyperthyroidism (thyrotoxicosis). Both are treated with radioiodine therapy I-131 to detect any area of residual thyroid tissue or tumour. Thyroidectomy is regularly performed on the malignant tumour^4^. Other promising technology was used, such as thermal ablative (TA), as alternative treatment options for thyroid diseases and has successfully succeeded in cancer treatment. The volume reduction rate (VRR) of the tumour exceeds 99%. TA procedures are known to be effective also in combination with radioiodine-131 treatment^5–7^. TA such as microwave ablation (MWA) use hyperthermia effects to destroy tumours through protein denaturation. The procedure is safe and effective in treating thyroid cancer. It provides innovative alternative therapy with minimal postoperative distress, shorter time of operation, and hospitalization stay, which can considerably increase the patients’ lives^8^. However, some complications were reported during MWA including, hoarseness, skin burning sensation, and haemorrhage^9,10^. Laser ablation (LA, λ = 1064 nm and power = 3–4 Watts) under ultrasound guidance is used for thyroid cancer treatment and accomplished high therapeutic outcomes with less cost and complications than surgical operation^10^. The main drawback of LA is the high temperature (≈ 110 °C), which could cause tissue burning, which may result in wound healing delay^11^. Radiofrequency ablation (RFA) has a comparable outcome with complications less than 4.5%^12^, with effective and safe treatment when surgical intervention cannot be executed^8,13,14^. High-Intensity Focused Ultrasound (HIFU) is used to eradicates tumours cells by thermal coagulation (≈ 60 °C) while sparing the superficial tissues^15^, with limited complications^16^. TA main complications are thermal damage, which could be relieved in a short period. The overall safety of the TA techniques is primary tumour location dependant. Operator experience was reported to be crucial in procedural complications^8^. However, in this study, all thyroid cancer patients underwent surgical operation (thyroidectomy) followed by radioiodine therapy as standard protocol^10,17^. Radioiodine provides sufficient curative outcome, including patients with metastatic thyroid cancer^18^.

Concerning thyroid disorders and the associated theranostic clinical applications, radioactive iodine-131 (Z = 53, T_1/2_ = 8.02 days) has now been used as an unsealed source for a period of in excess of seven decades, offering 90% beta emission (606 keV, range in tissue = 0.8 mm) and 10% gamma emission (364 keV)^19^. ^131^I cancer ablation (for total thyroidectomy), using activities ranging between 1110 to 7400 MBq (30–200 mCi) provides an excellent treatment option, an exact and targeted therapy with minimal side effects, allowing preservation of healthy tissues and cells beyond the tumour region, with monitoring of the disease using thyroglobulin serum levels^20,21^. In addition, thyroid remnant ablation by radioactive iodine (RAI) during thyroid hormone withdrawal(THW) has a high rate of complete ablation (75–90%) when high activity (3.5–3.7 GBq) is used^20^.

Exposure to ionizing radiation from different sources (fallout, the Chernobyl accident, medical exposures, etc.) have lead to cases of cancer, high energies and penetration giving rise to DNA damage^22^. Medical personnel (medical physicists, technologists, physicians, and nursing staff) interact with RAI-treated patients, after administration of radioiodine, including the entire period of hospitalization; hence they are exposed to ionizing radiation emitted by the patients. Radiation exposure depends on the time, distance, shielding, and workload; thus, staff exposure is variable. Recent studies have shown medical physicists, technologists, and nurses receiving annual doses of 604-, 680-, and 1000 µSv respectively^23–25^. Abu-Khaled et al^26^ reported respective annual shallow and deep dose values at various locations, including at the patient bed (226- and 175 mGy), bathroom (94 and 72 mGy) and visitor reception (12 and 10 mGy). Reported effects include that ionizing radiation occupational exposure induces DNA damage in the leukocytes of nuclear medicine employees^27^.

Unsealed radiopharmaceuticals such as ^131^I are frequently used in the nuclear medicine department for therapeutic purposes, potentially giving rise to significant occupational doses, up to 7.7 mSv per year in the recorded study of Bitar et al^24^. Thus, it is essential to ensure that staff receive minimal occupational dose from external and internal incorporated radioiodine, the latter due to inhalation of radioactive iodine due to its volatile nature (airborne iodine as an aerosol, CH_3_I and iodine vapour (I_2_))^28–30^. Miszczyk et al^31^ have reported staff radioiodine incorporation at a nuclear medicine department of up to 217 ± 56 Bq. Krajewska & Pachocki^32^ measured the mean and range of radioiodine activity (Bq) in the thyroid of the personnel at nuclear medicine staff were 83 (70–250), 280 (70–4000), and 275 (70–1000) for technical personnel, nuclear medicine personnel, and hospital service personnel, respectively. Therefore, the measurement of occupational radiation exposure and assessment of the associated biological risk is crucial in seeking to ensure staff are working in a safe environment. The objectives of this study has been to evaluate patient and occupational exposures arising from therapeutic radioiodine procedures, also measuring ambient doses and estimating the radiation risk.

## Materials and methods

### Radiation dose measurements

Occupational exposure was measured for seven personnel involved in the ^131^I radioiodine treatment of 182 patients receiving thyroid cancer therapy (138 (75.8%) female and 44 (24.2%) male) and 24 patients receiving treatment for hyperthyroidism (3 (12.5%) males and 21(87.5%) females), all within a one year period at King Faisal Specialist Hospital and Research Center (KFSHRC) (Tables 1 and 2). In the Kingdom of Saudi Arabia (KSA) KFSH&RC is one of the leading referral centers for thyroid cancer treatments with radioiodine. The Ethics and Research Committee at KFSH&RC center approved the research (RAC: 2201283), and written inform consent was obtained from each patient's prior data collection. Patient data include age, weight, and body mass index (BMI, kg/m^2^); administered activity and exposure geometry were also detailed.

**Table 1:** Mean, ± Sd and range of patient demographic data in thyroid cancer by using Iodine-131.

Gender	No. patient	Age (y)	Height (m)	Weight (kg)	BMI (kg/m^2^)
Male	44	(44.3 ± 14.9) (21–77)	(1.8 ± 0.1) (1.56–1.89)	(94.1 ± 16.3) (67.9–129.0)	(30.7 ± 4.7) (22.0–40.7)
Female	138	(42.7 ± 13.7) (16–81)	(1.7 ± 0.1) (1.3–1.7)	(72.3 ± 14.9) (33.7–112.6)	(29.5 ± 5.9) (15.4–43.4)
Total	182	(43.5 ± 14.3) (16–81)	(1.75 ± 0.1) (1.3–1.89)	(83.2 ± 15.6) (33.7–129)	(30.1 ± 5.3) (15.4–43.4)

**Table 2:** Mean, ± Sd and range of patient demographic data in hyperthyroidism by using Iodine-131.

Gender	No. patient	Age	Height (m)	Weight (kg)	BMI (kg/m^2^)
Male	3	(48.0 ± 25.16) (19–64)	(1.72 ± 0.02) (1.7–1.73)	(77.95 ± 0.1) (77.9–78.0)	(26.51 ± 0.63) (26.06–26.96)
Female	21	(35.24 ± 10.2) (20–61)	(1.58 ± 0.05) (1.5–1.67)	(70.1 ± 16.48) (55.1–97.0)	(27.97 ± 6.5) (22.1–39.4)
Total	24	(41.62 ± 17.68) (19.0–76)	(1.65 ± 0.04) (1.5–1.73)	(74.03 ± 8.3) (55.1–97.0)	(27.2 ± 3.57) (22.1–39.4)

### Patient demographics and radioiodine administration

**Figures 1:**
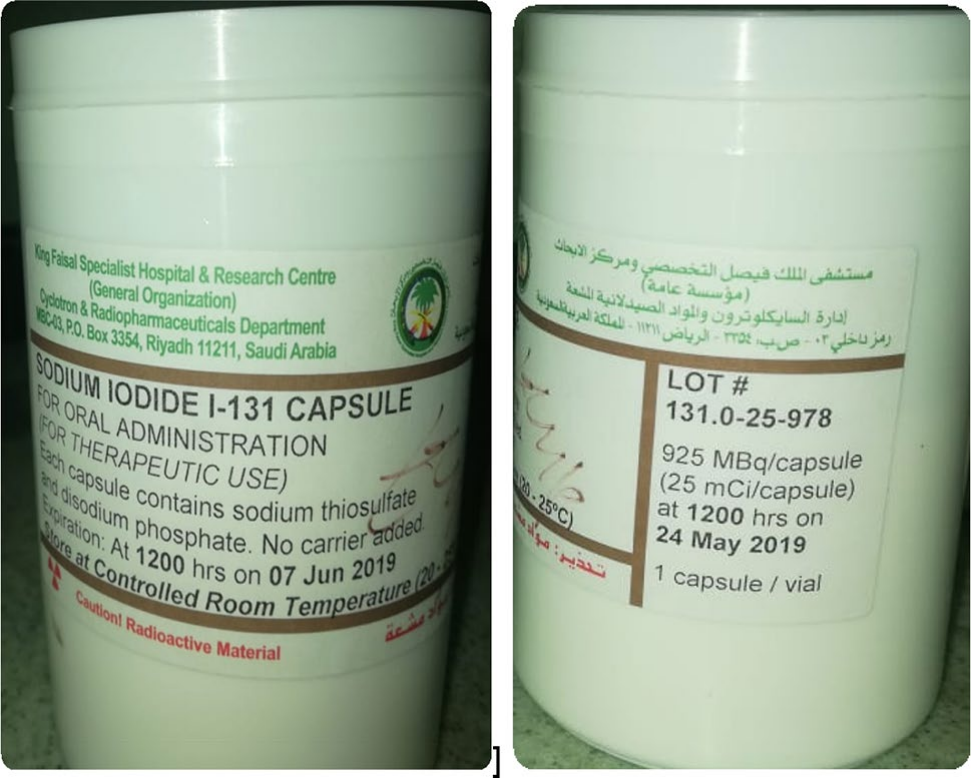
(**A**,**B**) Radioactive iodine.

At this center radioiodine therapy is via oral administration of capsules. Each radioiodine capsule also contains sodium thiosulphate (Na_2_O_3_S_2_) and disodium sulfate (Na_2_O_4_S) (Fig. 1). ^131^I was administered in the hospital at the patient bed of each patient. In this, in respect of potential spills etc., the patient was asked to sit at a table covered with adsorbent pads; the floor beneath the patient was also covered by adsorbent pads. The ^131^I is administered in capsules delivered to the patient in a shielded container (3.0 cm Pb). Post-administration, the patient is advised to drink several glasses of water in order to clean the mouth of any potential early release of the ^131^I. At this center the practice is that thyroid uptake and imaging are carried out within 24 h of administration. In Saudi Arabia, the dose limit concerning patient discharge is 18 µSv/h at a one meter distance with safety and protection instructions. The average hospitalization time is three days upon receiving the ^131^I capsules, according to the international commission on radiological protection (ICRP), and the international atomic energy agency (IAEA)^33,34^.

### Isolation ward

A nuclear medicine department that makes use of ^131^I as a radioactive source for treatment needs patient waste product to be drained into a separate waste management facility, referred to as a delay tank. There will be a significant amount of radioactive waste generated by the patient. Within three days of administration it has been estimated that almost 70% of ^131^I can be excreted in urine from the patient^35^. At KFSH&RC, patients treated with ^131^I remain in isolation for three days, six isolation rooms being available for this, to protect staff and members of the public from radiation exposure. During the isolation period, waste is drained into isolated delay containers at the department. All parts of the radiation protection policy are carried out in accord with national and international recommendations for occupational and public dose limits. In regard to the maximum annual exposure for staff working with radiation in this particular nuclear medicine department, annual effective dose may be anticipated to be ≥ 2.0 mSv (i.e. ≥ 10% of the permissible dose limits, applicable to the particular medical physicists, physicians, technologists, and nurses). For the purpose of record, the limit is recognized to be 20 mSv/year, 100 mSv for five years, with 50 mSv being the maximum dose for a single year. The annual dose for members of the public (visitors and comforters) is limited to 1.0 mSv^36,37^. In the nuclear medicine department, infants and children are not allowed access to the ward area. At this time, there are no dose limits for the patient for medical procedures.

### Staff incorporated radioiodine assessment

Occupational exposure to radioiodine may result in the accumulation of radioiodine in the thyroid. In particular, if staff work in a controlled area then monitoring of the thyroid uptake of staff is recommended, the recorded dose needing to be kept for at least 30 years^38^. The procedure is performed in a sitting position with the detector field of view placed at the neck level. Thyroid activity from incorporated radioiodine was quantified using thyroid uptake measurements for all staff (one medical Physicist and six nursing staff). Thyroid bioassay for staff is used rather than urine samples analysis because it is more accessible, the result is faster, cheap and can be performed on a self-screening basis. Urine analysis is an alternative technique but is require more time and cost for monitoring incorporated radioiodine^35^. Additionally, ambient doses have been measured at the wall, 1.5 m height. Conversely, for emission from patients during hospitalization, exposure has been estimated based on measurement at 30-, 100-, and 300 cm using calibrated survey meters (Victoreen 451P, Fluke Biomedical).

The ^131^I administered activity (AA) was calculated using the following Eq.^39^:

1 $$A (MBq)=\frac{23.4\times m(g)\times D(Gy)}{U\times T}$$

where 23.4 conversion factor, m is the thyroid mass in gram, U the thyroid dose uptake for 24 h, D absorbed dose of ^131^I and T the effective half-life of radioiodine.

MIRD dose was used for thyroid dose evaluation of the radioiodine activity requisite to accomplish a definite recommended absorbed dose (D) for the thyroid remnant lesion according to Eq. 2^40^.

2 $$D = \frac{\tilde{A} \times S\times {m}_{t}}{{m}_{t}}$$

where $\tilde{A}$ is cumulative activity, ${m}_{t}$ is the thyroid mass or remnant lesion in gram (reference mass is 20.7 g), S is dose to thyroid or remnant lesion from unit cumulated radioactivity (S-factor).

### Occupational exposure dosimetry and shielding

The current practice mobile shield of the lead equivalent 3 cm is used for regular practice. Occupational exposure for radioiodine treatment personnel was measured using two groups of thermoluminescent detectors (TLD-100). TLD-100 badge worn on the collar level.Extremity doses were measured using ring dosimeters placed on the dominant hand of the operator. TLD 100 (Harshaw-Bicron, USA) was used in this study, acknowledging their ability to make accurate radiation dose measurements for a wide range of dose from 10^–7^ Gy to 12 Gy^41^. Low fading is an essential characteristic of personal dosimetry, enabling dose measurement to be made at two-month intervals in routine departmental work. Calibration of the TLDs was made using a ^137^Cs source at the Secondary Standard Dosimetry Laboratory (SSDL), also located at the KFSH&RC. The TLD cards that are used provide for occupational calibration exposure (mSv) in terms of skin dose (Hp (0.07)) and deep dose (Hp(10)). All TLD signals were acquired using a TLD reader (Harshaw 6600) (Harshaw-Bicron Company, USA). The time–temperature profile adopted consisted of 100 °C preheating and signal acquisition up to 240 °C at a heating rate of 10 °C/s. Pre and post-irradiation annealing were applied for all TLDs batch using an automatic Oven (TLDO; Germany), settings being in accord with the manufacturer recommendations.

### Ambient dose and patient room measurement

Ambient dose measurements were also performed using calibrated TLD-100 detectors. These were placed at particular key locations around the radioiodine therapy department, including the nursing station (reception) and corridor of the department onto which all of the patients room opened. In addition, dose measurements were also carried out in the patient rooms, at the following locations: toilet, bed and basin, use being made of a survey meter (Victoreen 451P, Fluke Biomedical).

## Results

The results of this study represent a total of 206 patients, 182 (88.3%) and 24 (11.7%) receiving iodine therapy for thyroid cancer and hyperthyroidism, respectively. The incidence of thyroid cancer in female is higher compared to the male group. The incidence in female is 10% and 66% for hyperthyroidism and thyroid cancer, respectively. Similar findings were reported in previous studies^1,3,23,42,43^. Patient demographic data (age (y), weight (kg) and height (m)) showed the majority of patients to be overweight and obese, with an average BMI (kg/m^2^) of 30.1 ± 5.3 and of range between 15.4 to 43.4. The mean and range of administered activity (AA, MBq)) for thyroid cancer treatment were 4243.7 ± 2021.4 (1668.9–8066.0) (Table 3). The mean and range of AA (MBq) for hyperthyroidism were 1507.9 ± 324.1 (977.9–1836.9) (Table 4). The annual occupational doses were 1.2 mSv. The ambient doses at the isolation rooms after cleaning, also the corridors, were 0.2 mSv. Staff incorporated radioiodine was found to be below the lower limit of detection. Tables 3 and 4 show the dose rate measurements at different distances from the patients over a three days period, with and without a shielding barrier.

**Table 3:** Mean, ± Sd and range of patient demographic data in thyroid cancer by using Iodine-131.

Gender	Administered activity (MBq)	Dose rate 1st day (µSv/h)				Dose rate 2nd day (µSv/h)		Dose rate, 3rd day (µSv/h)	
		30 cm	100 cm	300 cm	Behind bed shield	30 cm	100 cm	30 cm	100 cm
Male	4503.0 ± 2046.6 (1825.5–8066.0)	598 ± 309 (150–1380)	114 ± 56 (25–230)	4 ± 2 (0.1–13)	4 ± 2 (0.1–9)	204 ± 86 (65–380)	56 ± 28 (12–137)	77 ± 46 (0.0–180)	(15 ± 6) (0.0–25)
Female	3984.3 ± 1996.2 (1512.2–8066.0)	499 ± 311 (100–1370)	(99 ± 53) (25–240)	4 ± 2 (0.1–12)	4 ± 2 (0.3–12)	165 ± 82 (35–640)	41 ± 23 (11–150)	51 ± 33 (6–150)	11 ± 5 (1–30)
Total	4243.7 ± 2021.4 (1512.2–8066.0)	5490 ± 3150 (1250–1380)	(107 ± 5.5) (25–240)	40 ± 20 (0.1–13)	4 ± 2 (0.1–12)	185 ± 84 (50–640)	49 ± 26 (11–150)	64 ± 39 (0–180)	13 ± 5.5 (0.0–30)

**Table 4:** Mean, ± Sd and range of patient demographic data for Hyperthyroidism by using Iodine-131.

Gender	Administered activity (MBq)	Dose rate first day (µSv/h)				Dose rate second day (µSv/h)		dose rate discharge day (µSv/h)	
		30 cm	100 cm	300 cm	Behind bed shield	30 cm	100 cm	30 cm	100 cm
Male	1187.21 ± 33.1 (1149.2–1209.9)	154 ± 41.2 (120–200)	29 ± 12 (17.6–41)	1.9 (1.0–3.0)	2.3 (1.0–5.0)	89 ± 26 (65–117)	18 ± 5 (15–24)	38 ± 12 (24–46)	(9 ± 4) (5–12)
Female	1190.9 ± 120.9 (806.6–1313.5)	147 ± 44 (65–240)	31 ± 8 (17–45)	2.6 (1.0–9.0)	2.1 (1.0–7.0)	56 ± 23 (0.0–100)	13 ± 5 (0.0–22)	22 (0.0–70)	5.8 ± 4.8 (0.0–15)
Overall	1189.1 ± 77.0 (806.6–1313.5)	1510 ± 430 (65–240)	30 ± 10 (17–45)	2.3 (1.0–9.0)	2.2 (1.0–7.0)	73 ± 25 (0–117)	16 ± 5 (0.0–24)	(27 ± 17) (0–70)	7.4 ± 4.4 (0.0–15)

## Discussion

**Figure 2:**
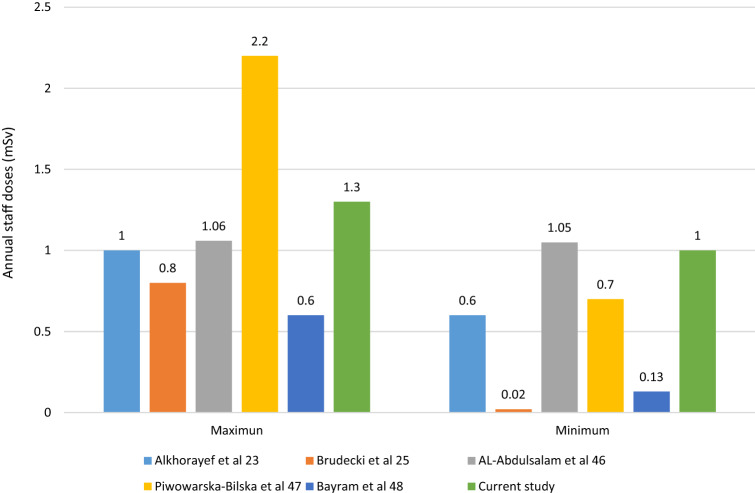
Occupational exposures compared to that recorded in previous studies.

For particular thyroid disorders, Iodine-131 therapy represents a highly effective theranostic radiopharmaceutical, with an ability to provide safe treatment as a result of the significant beta-decay component. Conversely, for nuclear medicine personnel, gamma emission represents the primary source of external exposure. Administration is made of significant radioactivity, typically with radioiodine treatment activities ranging from some 1700- to in-excess of 8000 MBq per patient. With this, patients effectively become an open-source of radiation exposure to surrounding personnel and to the environment, the latter through body fluids excretion. Patient dose is monitored daily to ensure dose reduction accords with treatment planning. In respect of hospital protocol, seeking to ensure that family members are well protected, patients are typically released when the dose-rate falls to below 18 µSv/h at a distance of one metre. The practice varied considerably regarding the release of patients from the hospital after radioiodine therapy. For example, in Japan, 500 MBq or <30 µSv/h at 1 m distance while for Germany it is 250 MBq or < 3.5 µSv/h at 1 m distance, and in the USA it is 1200 MBq or < 70 µSv/hr at 1 m distance. The IAEA and ICRP recommended that the release of patients treated with radioiodine should be decided on an individual basis^33,34^. Although in present study staff thyroid doses were found to be below the detection limit, others have reported that measured activities have ranged from 5.0 ± 2 Bq to 217 ± 56 Bq^44^,^25,45^. The average occupational dose and range (in mSv) from this study were found to be 1.2 (1.0–1.3) mSv per year, while the ambient dose was found to be 0.2 mSv per year, occupational doses being higher than most values reported in the literature (Figure 2). Previously reported that occupational exposure is patient mobility dependent. Self-caring patients expose the staff to lower doses compared to dependent patients^45^. Of note is that this occupational exposure includes only radiation doses resulting from the working environment. All other sources of other exposure have been excluded, including background radiation and medical exposure as a patient. In present study, the radiation-induced cancer risk from occupational exposure has been found to be well below the annual exposure limit of 20 mSv/year. Figure 2 showed the annual occupational exposure in previous studies^23,25,46–48^. The wide variation of occupational exposure attributed to the variation in the radioisotopes used in the department, radiation protection measures and the type of activity conducted by nuclear medicine personnel. Physicians received the least doses while technologist and nurses received the highest doses. Particular guidelines, including in regard to time, distance and shielding will help to ensure that the annual dose is kept well below the limit^26^.

## Conclusions

In the light of current practice, albeit applied to a relatively high workload, staff exposures were found to be below 1.2 mSv, the annual dose limits being 20.0 mSv. Occupational doses have been found higher than most of previously published studies. Proper patient isolation is an essential factor in staff radiation dose reduction. Current practice is noted to comply with international guidelines, the adoption of radiation safety recommendations being found highly effective in control of staff and public exposures.
